# Control of Directionality in *Streptomyces* Phage φBT1 Integrase-Mediated Site-Specific Recombination

**DOI:** 10.1371/journal.pone.0080434

**Published:** 2013-11-21

**Authors:** Lin Zhang, Binyan Zhu, Ruixue Dai, Guoping Zhao, Xiaoming Ding

**Affiliations:** 1 Department of Microbiology and Microbial Engineering, School of Life Sciences, Fudan University, Shanghai, China; 2 Key Laboratory of Synthetic biology, Institute of Plant Physiology and Ecology, Shanghai Institutes for Biological Sciences, Chinese Academy of Sciences, Shanghai, China; 3 Shanghai-MOST Key Laboratory of Health and Disease Genomics, Chinese National Human Genome Center at Shanghai, Shanghai, China; 4 Department of Microbiology and Li Ka Shing Institute of Health Sciences, The Chinese University of Hong Kong, Prince of Wales Hospital, Shatin, New Territories, Hong Kong SAR, China; Saint Louis University, United States of America

## Abstract

*Streptomyces* phage φBT1 integrates its genome into the *attB* site of the host chromosome with the *attP* site to generate *attL* and *attR*. The φBT1 integrase belongs to the large serine recombinase subfamily which directly binds to target sites to initiate double strand breakage and exchange. A recombination directionality factor (RDF) is commonly required for switching integration to excision. Here we report the characterization of the RDF protein for φBT1 recombination. The RDF, is a phage-encoded *gp3* gene product (28 KDa), which allows efficient active excision between *attL* and *attR*, and inhibits integration between *attB* and *attP*; Gp3 can also catalyze topological relaxation with the integrase of supercoiled plasmids containing a single excision site. Further study showed that Gp3 could form a dimer and interact with the integrase whether it bound to the substrate or not. The synapse formation of *attL* or *attR* alone with integrase and Gp3 showed that synapsis did not discriminate between the two sites, indicating that complementarity of central dinucleotides is the sole determinant of outcome in correct excision synapses. Furthermore, both *in vitro* and *in vivo* evidence support that the RDFs of φBT1 and φC31 were fully exchangeable, despite the low amino acid sequence identity of the two integrases.

## Introduction

Bacteriophages typically insert their genomes into host chromosomes *via* integrase-mediated site-specific integration between two sites of *attB* and *attP* from bacteria and phage, respectively, and form *attL* and *attR* sites to establish a lysogenic state. Under inducing conditions, the phage genome is eliminated *via* site-specific excision between *attL* and *attR* to convert into the lytic life cycle [1], [2]. The phage encoded integrase protein is required for both integration and excision [3], [4], however, this process is highly unidirectional and controlled by a recombination directionality factor (RDF or Xis) [1], [3], [5], [6], [7].

Phage-encoded site-specific integrases have been classified into two major groups: tyrosine and serine recombinases, which contain tyrosine or serine to attack DNA substrates in the active sites of the proteins [5]. The best studied member of the tyrosine recombinase group is that from *E.coli* phage λ [8], which has been extensively investigated both biochemically and structurally for decades [8], [9], [10], [11], [12], [13]. The recombination sites for λ integrase are quite different, as the *attB* site is short (25 bp) and simple, while the *attP* site is relatively large (240 bp) and contains multiple binding sites for integrase, Xis, IHF (integration host factor) and Fis (factor for inversion stimulation) [14], [15]. Xis is the master regulator of λ recombination, and three Xis-binding sites have been found in *attP* [16]. DNA bending catalyzed by Xis promotes the formation of an excisive intasome, but inhibits the formation of the integrative intasome [13].

In contrast, the mechanism of serine recombinases is less well understood, and the γδ resolvase is a member of the better known serine recombinases [5], [17], [18]. A number of phage-encoded integrases which were classified into the large serine recombinase subgroup [19], typically contain a large C-terminus which may be involved in DNA binding and directionality control [5], [20]. The best studied of these integrases *in vitro* are those of the *Streptomyces* phages φC31 [21], φBT1 [22], [23] as well as TG1 [24], and the mycobacterial phages Bxb1 [25] and φRv1 [4]. In each of these cases, the recombination sites of *attB* and *attP* are simple and short (less than 50 bp), and contain central dinucleotides of crossover sites which may control the polarity of the recombination [26]. To date, RDFs of phage TP901-1 [27], Bxb1 [3], φRv1 [4] as well as φC31 [1] have been identified and actively allow excision between *attL* and *attR*, and inhibit integration between *attB* and *attP*. A DNA binding assay of φC31 and Bxb1 Xis proteins strongly supported the view that Xis interacts with the *att*-bound integrases to change the conformation of the complexes that favor proceeding to excisive recombination [1], [3], [5]. Furthermore, two new reports on large serine recombinases, one of a single-molecule analysis of Bxb1 recombination revealed the molecular bearing mechanism of DNA strand exchange [28], and the second on φC31 integrase using hybrid “*phes*” recombination sites proposed a gated rotation mechanism [29]; both strongly support the “subunit rotation” model for exchanging DNA strands [30].

*Streptomyces* phage φBT1 is a temperate phage related to φC31 that integrates its genome into *SCO4848* coding a putative integral membrane protein of *Streptomyces coelicolor* [31]. We previously established a highly efficient site-specific *in vitro* recombination system based on purified φBT1 integrase, and determined the minimal sites of *attB* and *attP* [23]. The biochemical mechanisms underlying synapsis, strand cleavage and rejoining were further studied, and a model in which two alternative pathways can lead to synaptic complex formation of integration was proposed [22]. Furthermore, φBT1 integrase-based methods have also been widely used in various *Streptomyces* strains [31], [32], [33], [34], mammalian cells [35] and *in vitro* DNA assembly [36]. However, the directionality control factor of the φBT1 recombination system has not been identified to date. Here, we aim to characterize the RDF that regulates the directionality of recombination catalyzed by φBT1 integrase. The RDF protein of φBT1, which is encoded by phage *gp3* gene, was sufficient to activate *in vitro* excisive recombination and inhibit integrative recombination.

## Materials and Methods

### Strains, bacteriophages and plasmids

*E.coli* and the *Streptomyces* strains used in this study are described in Table S1 in File S1. Construction of phagemid φXD101 was as follows: A 3037 bp PCR product containing *gp15* to *gp22* of phage φC31 using primers 5′-AC*TCTAGA*GCCGAAGGCGCCACGCAA-3′ and 5′-GC*GGATCC*GTCGCTGGGTGGACGTAC-3′ was amplified and digested with XbaI and BamHI, and inserted into the XbaI-BamHI-cleaved plasmid, pSET152, to generate a gene-targeting vector pDXM101. Plasmid pDXM101 was then introduced into *Streptomyces coelicolor* strain J1929 containing a φC31 lysogen by conjugation from *E.coli* strain ET12567/pUZ8002. After selected on Apramycin agar, the positive colonies, mixed with both homologous recombinants targeting φC31 prophage and integrative exconjugants, were pooled. Spores of those colonies were used for the burst of phages, plated onto soft agar with indicator strain (spores of wild type *Streptomyces coelicolor* strain J1929) to yield plaques. The apramycin resistant phage was then obtained by transformation of the isolated phage DNA into *E.coli* strain DH10B and selected with antibiotics. That resulting phagemid, designated φXD101, can be maintained in *E.coli* as a plasmid, and conjugated into *Streptomyces* as an active phage. The phage genes *gp23* to *gp28* were deleted during the process of homologous recombination followed by phage packaging to adapt the phage genome size. Construction of plasmid-phage φXD101(X02) and φXD101(X03) by the PCR-targeting method [37] was as described in the Results and Discussion. Details of the construction of other plasmids and primers used in this study are described in Table S1 and Table S2 in File S1.

### Protein expression, purification and crosslinking

Expression and purification of φBT1 and φC31 integrase were as described in our previous work [22], [23]. For φBT1 excisionase (Gp3), the *gp3*-φBT1 gene (accession number AJ550940.2) was chemically synthesized and cloned into expression vector pET-28b (+) to generate pET-28-Bxis, that now carries a poly-His tag fused at the N-terminus of the *gp3*-φBT1 gene. In addition, the φC31 excisionase, the *gp3*-φC31 gene, was PCR amplified from phage φC31 and cloned into pET-28b (+) (see Table S1 in File S1). The procedures for protein expression, purification [23] and crosslinking [38] were performed as described previously.

### *In vitro* recombination and relaxation assay

Standard recombination was carried out in a reaction mixture (10 µl) containing 10 mM Tris-HCl at pH 8.0, 100 mM KCl, 5% glycerol and integrase (270 nM) [23] with or without excisionase (0.25 µl),except where otherwise indicated. The final concentration of monovalent cations (K^+^ and Na^+^) was 150 mM. Reactions using linear DNA substrates were incubated at 30°C for 30 min (or as indicated) and were terminated by heat inactivation at 75°C for 10 min or treated with proteinase K at 55°C for 30 min; products were separated by electrophoresis on agarose gels (0.8%). Reactions using supercoiled plasmids for the quantification of recombination efficiency were treated with proteinase K at 55°C for 30 min followed by transformation into *E.coli* strain DH10B.

Topological relaxation assays were performed with supercoiled plasmids containing *attB* (plasmid pZLB00), *attP* (plasmid pZLP00), *attL* (plasmid pZL5816), and *attR* (plasmid pZL5817) (see Table S1 in File S1). The DNA substrates were incubated with φBT1 integrase and (or) excisionase for 1 hour. Reactions were carried out similar to the *in vitro* recombination assays; however, the reactions were terminated by heat inactivation at 75°C for 10 min and separated by electrophoresis on agarose gels (0.8%) in TAE buffer, and visualized by post-staining with ethidium bromide (EtBr). The bands between the supercoiled DNA and relaxed circles displayed a “ladder” of closed circular DNA species, which were likely topoisomers with a decreasing number of knots.

### Electrophoretic mobility shift assay (EMSA)

FAM-labeling of DNA fragments was performed by PCR using the primer ZL93 5′-labeled with 5-FAM. *attB_212_* was amplified from pZLB00 using primers ZL95/ZL80 and then labeled using primers ZL93/ZL80; *attP_247_* was amplified from pZLP00 using primers ZL94/ZL82, and then labeled using primers ZL93/ZL82; *attL_306_* was amplified from pZL5819 using primers ZL95/ZL88 and then labeled with primers ZL93/ZL88; *attR_153_* was amplified from pZL5819 using primers ZL94/ZL80 and then labeled with ZL93/ZL80. Approximately 0.1 pmol (10 ng) of FAM-labeled *attB_212_*, *attP_247_*, *attL_306_* or *attR_153_* DNA were incubated with the indicated amounts of integrase in a binding mixture (10 µl) containing 20 mM Tris-HCl at pH 8.0, 100 mM KCl, 1 mM DTT, 5% glycerol and 500 ng of sonicated salmon sperm DNA; reactions were incubated at 30°C for 30 min and separated on 5% non-denaturingpoly-acrylamide gels in 1×TBE buffer at 4–10°C. DNA bands were visualized by fluorescence imaging using an FLA-9000 Starion Image Scanner (Fuji Film).

### Plaque assay

*S.coelicolor* spores of lysogen J1929 harbouring φXD101, φXD101(X02) and φXD101(X03) (1×10^6^ cfu) were incubated in 30 ml 2×YT medium at 30°C with shaking for 16 hrs. The cultures were filtered using a 0.45 µm filter membrane to obtain phage suspension, and 10 µl of the suspensions were pipetted onto Difco nutrient broth (DNB) agar with MgSO_4_ (10 mM) and Ca(NO_3_)_2_ (8 mM). The soft DNB top layers (containing spores of indicator strain J1929, 1×10^8^ cfu) were then added to each plate, and the plates were incubated overnight at 30°C to generate plaques.

## Results and Discussion

### Gp3 is the RDF in phage φBT1 recombination

*Streptomyces* phage φBT1 integrase-mediated site-specific recombination is highly efficient both *in vivo* [31], [35] and *in vitro* [22], [23], and has become a very useful tool in a variety of applications [32], [36]. However, the recombination directionality factor (RDF) of φBT1 has not yet been identified, and limits the further development of this system. The genome organization of phage φBT1 is highly similar to that of φC31, and the major gene products are closely related [31]. Previous studies have shown that the early phage protein, Gp3, from φC31 is an RDF of recombination which activates excision and inhibits integration [1], [39]. Amino acid sequence alignment of the Gp3 from φBT1 and φC31 showed that the two proteins shared 85% identity (see Figure S1 in File S1) [32]; it is likely that the Gp3 of φBT1 has the same function as Gp3-φC31 as an RDF. Thus, the *gp3*-φBT1 gene was chemically synthesized and cloned into the expression vector. Details of protein expression and purification are described in Materials and Methods. As shown in Figure S2 in File S1, Gp3-φBT1 was isolated with a purity over 95%, and was analyzed by both SDS-PAGE and gel filtration (data not shown).

**Figure 1:**
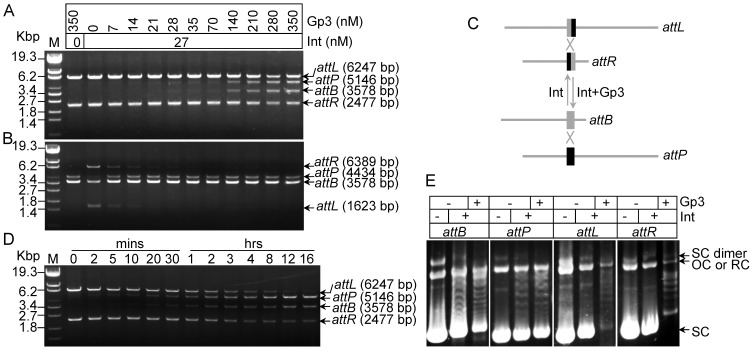
Gp3 with integrase catalyze excision in phage φBT1 recombination. (**A and B**) *In vitro* excision and integration recombination using linearized DNA in the presence of Gp3 and integrase. For the substrates of excision, plasmid pZL5813 was digested with KpnI to generate *attL* (6247 bp) and *attR* (2477 bp); the product sizes were predicted as 3578 bp for *attB* and 5146 bp for *attP*. For integration, pZL5812 was digested with HindIII to generate *attB* (3578 bp), and pZL5811 was digested with EcoRI to generate *attP* (4434 bp); the product sizes were predicted as 1623 bp for *attL* and 6389 bp for *attR*. Substrates were incubated with or without 27 nM integrase and varying concentrations (nM) of Gp3 for two hours. (**C**) Schematic diagrams of substrates used and the expected products in the excision and integration reactions shown in (A). (**D**) Time course of *in vitro* excision, and the substrates used were as shown in (A). The concentrations of proteins were 270 nM for integrase and 350 nM for Gp3. The reaction times are indicated. (**E**) DNA topological relaxation assays of *attB* (plasmid pZLB00), *attP* (plasmid pZLP00), *attL* (plasmid pZL5816), and *attR* (plasmid pZL5817) were performed with or without integrase or Gp3. The bands between the supercoiled and relaxed circles formed a ladder of closed circular DNA species that are probably topoisomers with a declining degree of superhelicity. The positions of supercoiled substrate DNA (SC), relaxed circles (RC), open circles (OC) and dimeric supercoiled DNAs (SC dimer) are indicated.

Then *in vitro* excision and integration assays were performed using linearized substrates. Considering the apparent binding affinities (*K_d_*) of integrase to the *attB*, *attP*, *attL* and *attR* sites observed at 60 nM in our previous study [22], approximately half of the concentration of integrase (27 nM) was used for primary assays (Figure 1A and 1B). The excision products (*attB* and *attP*) could be detected when an equal amount of Gp3 (28 nM) was added, and the productivities increased gradually with increasing amounts of Gp3. An obvious excision band was observed at 140 nM of Gp3; and inhibition of integration was detected when a little Gp3 (7 nM, 1/4 of Int) was added to the reaction (Figure 1B). This observation are consistent with the results observed in mycobacteriophage φRv1 excision, where 50% product formation was reduced by adding as little as 24 nM of RDF(concentration of integrase was 400 nM) [4]. However, it is different from that found in the φC31 recombination, that is, equal to or greater than 1∶2 Gp3 to Int was sufficient to inhibit integration [1]. Our data indicated that Gp3 monomer might interact with Int tetramer in the synaptic complex to inhibit integration.

**Figure 2:**
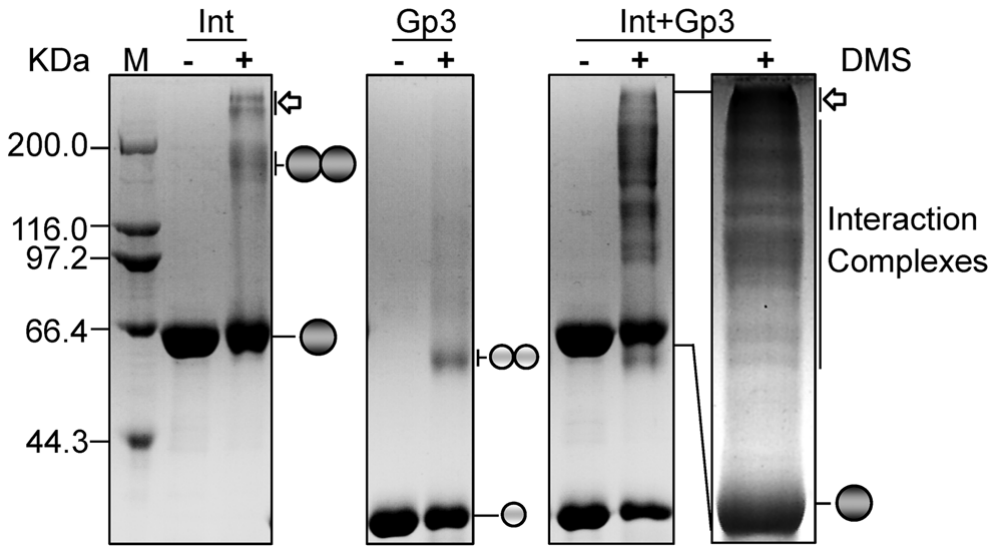
Crosslinking of φBT1 integrase and Gp3. Enzymes were incubated in the absence or presence of dimethyl suberimidate (DMS) and analyzed by SDS-PAGE (see Materials and Methods). The positions of integrase (Int) monomer and dimer, Gp3 monomer and dimer, as well as possible interaction complexes are illustrated. The bands indicated by hollow arrows could be higher order oligomers. M, protein molecular weight markers.

To determine the polymeric form of the Int and Gp3, crosslinking experiments were performed using purified proteins. As shown in Figure 2, both the Int and Gp3 formed dimers in solution; and when the two proteins were mixed together, a ladder of higher order oligomers was observed, indicating that a series of intermediate oligomers were formed. These results confirmed the protein-protein interactions of Int and Gp3 in solution; and indicated that these interactions could occur with different numbers of Int and Gp3 molecules, before obtaining the appropriate stoichiometry.

To investigate the kinetics of excision, the time course of *in vitro* excision was studied. The excision recombination showed relatively slower kinetics than that of integration [23], where 50% of products were observed in 0.5 hours; however, excision took 2 hours (Figure 1D). Furthermore, our previous study showed that integrase could catalyze the topological relaxation of supercoiled plasmids containing single integration sites (*attB* or *attP*) in a partner DNA-independent manner, but not for the excision sites (*attL* or *attR*) [22]. Therefore, topological relaxation assays of plasmids containing *attB*, *attP*, *attL* or *attR* were performed with or without Int or Gp3(Figure 1E). Topological relaxation catalyzed by φBT1 integrase using supercoiled *attL* or *attR* plasmids was observed following the addition of Gp3; and no inhibition of relaxation on supercoiled *attB* or *attP* plasmids was observed (Figure 1E), indicating that the presence of Gp3 had no effect on the DNA binding and cleavage activity of integrase on the single integration sites.

### DNA binding properties of Gp3

**Figure 3:**
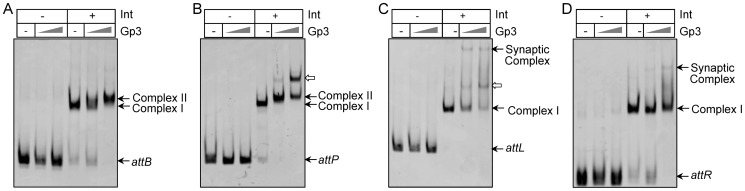
Gp3 acts as the RDF through interaction with integrase. Investigation of DNA binding properties of Gp3 with or without integrase (Int) using FAM-labeled *attB_212_* (A), *attP_247_* (B), *attL_306_* (C) and *attR_153_*(D). The Int concentration in the reactions was 135 nM, and concentrations of Gp3 were 175 nM and 1750 nM. The positions of free DNA, complex I, complex II and the synaptic complex are indicated. The bands indicated by hollow arrows in (B) and (C) could result from multiple Gp3 monomers interacting with the Int dimer at higher concentrations.

To test the ability of Gp3 to bind to substrates *attB*, *attP*, *attL* and *attR*, electrophoretic mobility shift assay (EMSA) was performed using FAM-labeled *attB_212_*, *attP_247_*, *attL_306_* and *attR_153_*. As shown in Figure 3, no DNA binding activity was detected when Gp3 was the only protein in the reactions, including at a high concentration of 1750 nM. Thus, similar to the RDFs of phage Bxb1 and φC31 [1], [3], the Gp3 of φBT1 had no DNA binding ability to the target substrates.

We further explored the properties of integrase bound to DNA in the presence of Gp3, since protein-protein interactions were observed in Figure 2. As shown in Figure 3A and 3B, integrase could bind to *attB* or *attP* to form “Complex I”; and after Gp3 was added, a slower migrating complex (complex II) was detected. This observation suggested that Gp3 might interact with integrase and form a stable complex to inhibit integration between *attB* and *attP* [1], [3], [5]. Nevertheless, the slower migrating complex (complex II) was not observed when substrates *attL* or *attR* were tested(Figure 3C and 3D); this result was consistent with the DNA binding properties in Bxb1 excision, where the RDF of Bxb1 might bind weakly to the *attL/R*-Int complexes and fail to be detected during gel electrophoresis [3]. However, slower migrating complexes were detected when integrase and Gp3 of φC31 were incubated with *attL* or *attR* [1]. It is interesting that although the RDF for φBT1 shared much higher identity of the amino acids sequences with φC31 than that of mycobacteriophage RDF for Bxb1, the effects on the conformations of *attL/R*-Int complexes may be diverse, or scarcely show any association with similarity of the amino acids sequences.

### Complementarity of central dinucleotides is the sole determinant of recombination outcomes in the correct excision synapses

**Figure 4:**
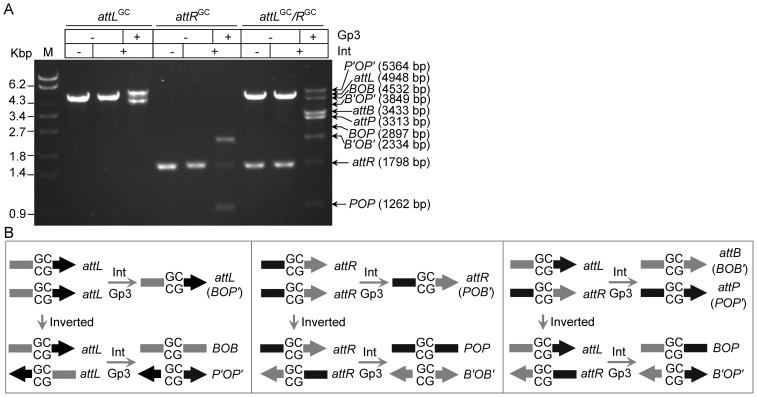
Substrates with palindromic central dinucleotides participate in *attL/attL* and *attR/attR* excision. (**A**) Excision reactions were performed using *attL* and *attR* with palindromic central dinucleotides. Plasmid pZLLR03 was digested with EcoRI and AgeI to generate linearized *attL^GC^* (4948 bp) and *attR^GC^* (1798 bp). The product sizes were predicted as 4532 bp for *BOB* and 5364 bp for *P′O P′*, 1262 bp for *POP* and 2334 bp for *B′OB′*, 3433 bp for *BOB′*(*attB*) and 3313 bp for *POP′*(*attP*), 2897 bp for *BOP* and 3849 bp for *B′OP′*. The positions of the substrates and series of products are indicated. The concentrations of proteins were 270 nM for integrase and 350 nM for Gp3. The reactions were terminated by proteinase K after incubation at 30°C for eight hours. (**B**) Schematic diagrams of the excision reaction shown in (A). Both *attL* and *attR* may form parallel and anti-parallel alignment, however, only correct alignments of substrates could participate in correct excision synapses, followed by excision.

**Figure 5:**
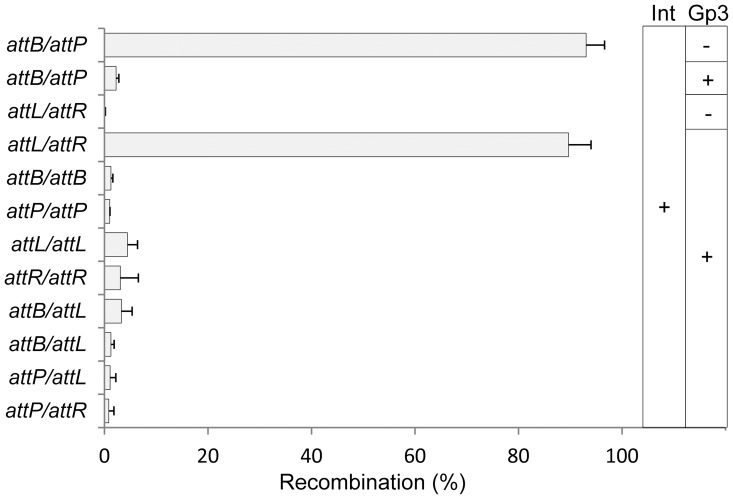
Quantification of *in vitro* excision efficiencies between two sites of *attB*, *attP*, *attL* or *attR*. *In vivo* detection of *in vitro* recombination products by blue-white screen; the four recombination targets were inserted into *lacZα* such that LacZ activity was abolished by recombination. The four target plasmids were pZLB00 (*atttB*), pZP00 (*atttP*), pZLL00 (*atttL*) and pZLR00 (*atttR*); the plasmids containing partner DNA were pZL5812 (*attB*), pZLP00 (*attP*), pZL5816 (*attL*) and pZL5817 (*attR*). After *in vitro* recombination, the plasmids were transformed into *E.coli* DH10B and plated on IPTG/X-gal medium; the white clones were produced by recombination. The substrate ratio of each combination was 5∶1 (partner to target). The data shown are average values of three reactions. The substrates used in each reaction and the relative recombination efficiencies (%) are indicated.

Interestingly, a very slow-migrating band was formed, as shown in Figure 3C and 3D, when single substrate *attL* or *attR* alone was incubated with Int and Gp3 in the reaction. However, this was not observed in the excision of φC31 [1]. This might be a consequence of *attL* or *attR* synapses with itself. It raised a question regarding *attL* or *attR* that each could be involved in recombination with itself. Thus, excision reactions were performed using substrates containing palindromic central dinucleotides (*attL^GC^* and *attR^GC^*). As shown in Figure 4, both *attL^GC^* and *attR^GC^* could participate in recombination with itself, and the unnatural products were generated by anti-parallel alignment of the substrates, i.e., *BOB* and *P′OP′* for *attL*, *POP* and *B′OB′* for *attR* (Figure 4B). Furthermore, there is the possibility of generating recombination products by parallel alignment of substrates; however, as shown in Figure 5, the relative recombination efficiencies of *attL/attL* and *attR/attR* were only 4% and 3%, respectively. Thus, these data supported the view that the slow-migrating band shown in Figure 3C and 3D was accumulation of synaptic complexes caused by anti-parallel alignment of *attL* or *attR* with itself, which contained 5′-GT central dinucleotides and further religation was suspended in the synapsis.

To gain further insights into the substrate alignments and formation of correct excision synapses in the recombination, *attL^GC^* and *attR^GC^* were incubated with integrase and Gp3 in one reaction. As shown in Figure 4, the natural excision products of *attB* and *attP* were detected; however, the unnatural products were formed with equal efficiency. Thus, the products for three forms of anti-parallel alignment of the substrates, i.e., *attL*/*attL*, *attR*/*attR* and *attL*/*attR*, were observed; and the product (*BOP* and *B′OP′*) for parallel alignment of *attL* and *attR* was detected with very low yield. Considering the low selectivity to the arm sequences of *attB* and *attP* in our previous study [22], it is believable that synapsis does not discriminate between *attL* and *attR* as represented in Bxb1 excision [40]. Thus, complementarity of central dinucleotides is the sole determinant in recombination outcomes once the correct excision synapses are formed. This property is consistent with that observed in excision recombination of Bxb1, φRv1 and φC31 [1], [20], [40].

### Substrate selection in the presence of integrase and Gp3

To address the substrate specificity of excision recombination, we used a quantitative *in vitro* excision assay. Reporter plasmid substrates were constructed in which the target sites were inserted in-frame into *lacZα* of plasmid pBC-SK(−). Following *in vitro* recombination with partner plasmid, the products were transformed into *E.coli* and plated on indicator media: white colonies indicated that recombination had occurred. As demonstrated in Figure 5, the frequency of white colonies was more than 90% when integration occurred between *attB* and *attP*; however, the efficiency was reduced to 2% in the presence of Gp3. Typical excision catalyzed by integrase and Gp3 between *attL* and *attR* gave 89% white colonies. No substantial amounts of white colonies were observed between other combinations of target and partner plasmids, although those between *attL*/*attL* (4%), *attR*/*attR* (3%) and *attB*/*attL* (3%) showed relatively higher efficiencies. However, it seems that excision of φBT1 showed more stringent substrate selectivity than that of φC31, where white colonies of 36% for *attL*/*attL* and 18% for *attR*/*attR* were observed [1].

### Cross-functional excision of integrase with Gp3 from φBT1 and φC31 respectively

Since the Gp3 proteins from phage φBT1 and φC31 shared 85% identity of amino acid sequences (see Figure S1 in File S1), it is reasonable to believe that the two proteins were exchangeable during *in vitro* recombination. The *gp3*-φC31 gene was then PCR amplified from phage φC31 and cloned into the expression vector, and the Gp3-φC31 was purified to near homogeneity (see Figure S2 in File S1).

**Figure 6:**
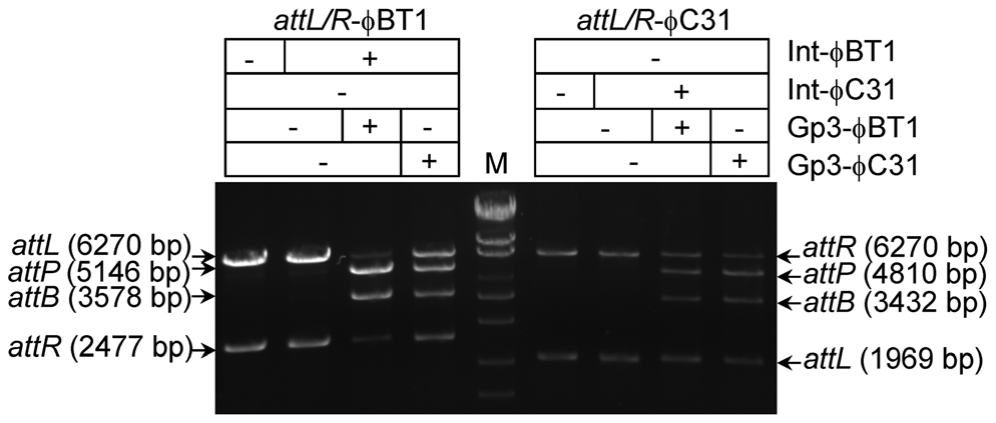
*In vitro* excision using Gp3 from φBT1 and φC31. Excision reactions were performed using *attL* and *attR* of φBT1 or φC31. *attL* and *attR* of φBT1 were obtained as described in Figure 1. For substrates of φC31, plasmid pZL5822 was digested with EcoRI to generate *attL*-φC31(1969 bp) and *attR*-φC31 (6270 bp); the product sizes were predicted as 3432 bp for *attB* φC31 and 4810 bp for *attP*-φC31. Pairs of excision substrates were incubated with their own integrase in the presence or absence of Gp3 from φBT1 or φC31. The concentrations of proteins used were 270 nM for integrase and 350 nM for Gp3. The reactions were terminated by proteinase K after incubation at 30°C for eight hours.

*In vitro* excision assays were performed both using linearized substrates of φBT1 and φC31. As shown in Figure 6, both Gp3-φBT1 and Gp3-φC31 could catalyze efficient excision between *attL*/*attR*-φBT1 or *attL*/*attR*-φC31 with their corresponding integrases. It is interesting that the φBT1 and φC31 integrases only showed 26% identity of amino acid sequences; their RDFs, however, shared very high identity (85%) and were proved replaceable through *in vitro* recombination assays (Figure 6). This suggested that the protein structures to interact with RDFs of φBT1 and φC31 integrases might be highly similar despite the low identity of amino acid sequences. Furthermore, the *Streptomyces* phage TG1 [24], [41] protein Gp25 (26.4 KD) shared 62% identity with Gp3-φBT1, and 60% of that with Gp3-φC31(see Figure S1 in File S1); suggesting the similar function of Gp25 in TG1 recombination. Furthermore, among four previous experimentally identified RDFs for large serine recombinases, Xis of the mycobacteriophage φRv1 (GpRv1584c, 8KD) [4] and lactococcal phage TP901-1 (Orf7, 7.5 KD) [27] are relatively small; Gp3 (27 KD) of *Streptomyces* phage φC31 [1] and Gp47 (28 KD) of mycobacteriophage Bxb1 [3] are larger. And they shared no identity of amino acid sequences, which is consistent with the diversity of RDFs [7].

### Gp3 of φBT1 could serve as the RDF in φC31 excision out of the host genome

To confirm the cross-functional excision property of integrase with Gp3 from φBT1 and φC31 *in vivo*, plaque assays of mutated phage φC31 were performed. Neither integration nor excision is typically essential for lytic propagation following phage infection; however, excision is required for productive lytic growth from a prophage. To test this, we constructed lysogen harboring φC31 with *gp3* deletion, wondering if the phage release will decrease and that mutation could be complemented by *gp3*-φBT1.

**Figure 7:**
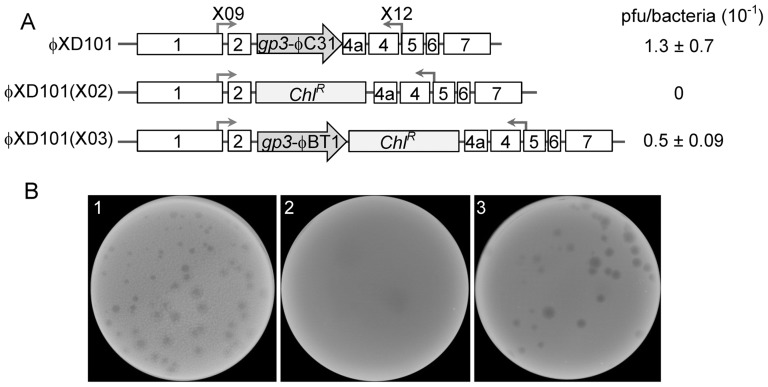
Plaque assay of mutated phage φC31. (A) Schematic representation of *gp3* and surrounding phage genes. Plasmid-phages φXD101(X02) and φXD101(X03) were derivatives of φXD101 by PCR-targeting technology. *gp3* of φXD101 was replaced by Chloramphenicol resistant gene (*Chl^R^*) in φXD101(X02), and further replaced by *gp3*-φBT1 and *Chl^R^* in φXD101(X03). The construction of φXD101(X02) and φXD101(X03) are described in Materials and Methods. Primers X09 and X12 were used for identification of the derivatives, and the results are shown in Figure S3 in File S1. (B) Plaque assays of phage φXD101, φXD101(X02) and φXD101(X03). The three plasmids were transformed into *Streptomyces coelicolor* strain J1929 by conjugation from *E.coli* strain ET12567/pUZ8002 and selected on Apramycin agar; the positive clones were isolated to burst phages. The phage suspension was then plated onto soft agar with spores of indicator strain to yield plaques. Plates 1, 2 and 3 were prepared using suspensions from J1929 harbouring φXD101, φXD101(X02) and φXD101(X03), respectively.

A φC31 derivative phagemid φXD101 was generated, which was maintained in *E.coli* as a plasmid, and conjugated into *Streptomyces* as an active phage. Details of the construction of φXD101 are described in Materials and Methods. As illustrated in Figure 7A, the *gp3*-φC31 gene of φXD101 was replaced by Chloramphenicol resistant gene (*Chl^R^*) to generate φXD101(X02) using the PCR-targeting system [37]; and further replaced by *gp3*-φBT1 and *Chl^R^* to obtain φXD101(X03). PCR analysis using primers X09/X12 confirmed the successful construction of the three plasmids (see Figure S3A in File S1). The plasmids were then introduced into *Streptomyces coelicolor* indicator strain J1929 by conjugation, and Figure S3B in File S1 shows the PCR verification of the positive exconjugants of wild-type *S.co* J1929 and harboring φXD101, φXD101(X02) or φXD101(X03).

As shown in Figure 7B, plaques were clearly formed when using phage suspension from strains harboring *gp3*-φC31 as well as *gp3*-φBT1; however, no plaques of the *gp3*-φC31 deletion construct were detected. The quantitative analysis of pfu/bacteria is shown in Figure 7A. The phenomenon that no plaques detected on the second plate was over expected. It seems like the RDF Gp3-φC31 or Gp3-φBT1, is required both for prophage excision and phage DNA replication, as described in Bxb1 RDF Gp47 [42]. This observation supported that Gp3-φBT1 shared identical function with Gp3-φC31 *in vivo*, could serve as the RDF in φC31 excision out of the host genome. Thus, Gp3-φBT1 and Gp3-φC31 are interchangeable in both *in vitro* and *in vivo* recombination; this could be a major concern when combining these two systems for genetic manipulation.

In conclusion, we have demonstrated that the phage-encoded protein, Gp3, is the RDF which controls the directionality of the reaction in φBT1 integrase-mediated site-specific recombination; and this function is realized by a protein-protein interaction with the integrase rather than direct binding to the substrates. Furthermore, the φBT1 integration system has been widely used for genetic engineering both *in vivo* [32], [33], [34], [35] and *in vitro* [22], [23], [36]; thus identification of the RDF (Gp3) reported here, could extend the potential utility of the φBT1 recombination system [32].

## Supporting Information

File S1 **Contains: Table S1, Table S2, Figure S1, Figure S2, Figure S3, Supplementary References.**
(DOCX)
