# Supplementary material for: Control of Directionality in Streptomyces Phage φBT1 Integrase-Mediated Site-Specific Recombination
Source: PLoS One. 2013 Nov 21;8(11):e80434. doi: 10.1371/journal.pone.0080434 (PMC3836970; doi:10.1371/journal.pone.0080434)
Supplement: File S1 — Contains: Table S1, Table S2, Figure S1, Figure S2, Figure S3, Supplementary References. (DOCX) [file pone.0080434.s001.docx]

Control of directionality in *Streptomyces* phage φBT1 integrase- mediated site-specific recombination

Lin Zhang, Binyan Zhu, Ruixue Dai, Guoping Zhao, Xiaoming Ding

**Supporting Information**

Inventory of Supporting Information

- **Table S1.** Bacterial strains, phages and plasmids used in this study.
- **Table S2.** Sequences of oligonucleotides.
- **Figure S1.** Amino acid sequence alignment of the putative Xis proteins from phages φBT1, φC31 and TG1.
- **Figure S2.** Purification of Gp3 of φBT1 and φC31 expressed in *E.coli*.
- **Figure S3.** PCR identification of the mutants.
- **Supplementary References.**

**Table S1.** Bacterial strains, phages and plasmids used in this study.

| Strains, phage and plasmids | Relevant characteristics | Ref. or sources |
| --- | --- | --- |
| ***E.coli*** |  |  |
| DH10B | *F^-^ endA1 recA1 galE15 galK16 nupGrpsL ΔlacX74 Φ80lacZΔM15 araD139 Δ(ara,leu)7697 mcrA Δ(mrr-hsdRMS-mcrBC) λ^-^* | Invitrogen |
| BL21(DE3) | *F^–^ompT gal dcmlonhsdS_B_(r_B_^-^m_B_^-^) λ(DE3 [lacI lacUV5-T7 gene 1 ind1 sam7 nin5])* | Novagen |
| ET12567/pUZ8002 | (F^-^ dam-13::Tn9 dcm-6 hsdMhsdR recF143 zjj-202::Tn10 galK2 galT22 ara14 pacY1 xyl-5 leuB6 thi-1) | [[1](#_ENREF_1)] |
| ***Streptomyces*** |  |  |
| J1929 | *S. coelicolor* M145 derivative*, ΔpglY* | [[2](#_ENREF_2)] |
| J1929/φC31 | J1929 lysogen harbouring φC31 | [[3](#_ENREF_3)] |
| J1929/φXD101 | J1929 lysogen harbouring φXD101 | This work |
| J1929/φXD101 (X02) | J1929 lysogen harbouring φXD101(X02) | This work |
| J1929/φXD101 (X03) | J1929 lysogen harbouring φXD101(X03) | This work |
| ***Phages*** |  |  |
| φC31 | Wild type phage, whole genome AC: AJ006589 | [[3](#_ENREF_3)] |
| φXD101 | Plasmid-phage, a derivate of φC31containing replication origin of *E.coli*, *Apra^R^*. | This work |
| φXD101(X02) | Plasmid-phage, a derivate of φC31containing replication origin of *E.coli*, *Apra^R^*; *gp3*::*Chl^R^*. | This work |
| φXD101(X03) | Plasmid-phage, a derivate of φC31containing replication origin of *E.coli*, *Apra^R^*, *gp3*::*gp3*(φBT1)*Chl^R^*. | This work |
| ***Plasmids*** |  |  |
| pET-28b(+) | Expression vector, T7 promotor, resistant to kanamycin. | Novagen |
| pZL5808 | PCR fragment(1790bp) of *int* with NdeI and XhoI from pFDZ15 in pET-28b(+). | [[4](#_ENREF_4)] |
| pZL5806 | PCR fragment of *int*-φC31 from pSET152 in pET-28b(+). | [[5](#_ENREF_5)] |
| pGH | Cloning vector which derivated from pBluescript II SK(+). | Generay |
| pGH-Bxis | pGH containing synthetic *gp3-*φBT1 gene. | This work |
| pET-28-Bxis | The *gp3-*φBT1 gene fragment from pGH-Bxis was digested with NdeI/XhoIand inserted into pET-28b(+) | This work |
| pMD-19T | T-cloning Vector, resistant to ampicillin | Takara |
| pMD-19T-Cxis | A 745 bpPCR fragment *containing gp3-*φC31 gene using primers X01/X02 was inserted intopMD-19T vector. | This work |
| pET-28-Cxis | A 738 bpDNA fragment *containing gp3-*φC31 from pMD-19T-Cxiswas digested with NdeI/XhoI and inserted into pET-28b(+) | This work |
| pZL5811 | pRT802 digested with *Eco*RI and self-ligated, the integrase gene was disrupted. | [[4](#_ENREF_4)] |
| pZL5812 | PCR fragment (886bp) *attB* from *S.coelicolor* genome in pMD19. | [[4](#_ENREF_4)] |
| pZL5813 | *attL* and *attR* in direct orientation after integration of pRT802 and pZL5812, resistant to kanamycin and ampicillin | [[4](#_ENREF_4)] |
| pZLB00 | A 45 bp wide-type *attB* cloned into pBC-SK(-), *lacZα, Chl^R^* | [[5](#_ENREF_5)] |
| pZLP00 | A 617 bp PCR fragment containing wild type *attP* cloned into pMD19, *Amp^R^* | [[5](#_ENREF_5)] |
| pZL5816 | A the integrase gene was disrupted.4258bp plasmid containing *attL*-φBT1 site,resistant to ampicillin. | [[4](#_ENREF_4)] |
| pZL5817 | A 3754bp plasmid containing *attR*-φBT1 site,resistant to kanamycin. | [[4](#_ENREF_4)] |
| pZL5819 | *attL* and *attR* in direct orientation after integration of pZLB00 and pRT802, *Chl^R^*, *Kan^R^* | [[5](#_ENREF_5)] |
| pZLLR03 | *attL^GC^*and *attR ^GC^* in direct orientation after  integration of pZLB03[[5](#_ENREF_5)]and pZLP03 [[5](#_ENREF_5)], *Chl^R^*, *Amp^R^*. | This work |
| pBC-SK(-) | Cloning vector, *lacZα*, *Chl^R^* | Stratagene |
| pZP00 | Wide-type *attP* cloned into pBC-SK(-), *lacZα, Chl^R^* | [[5](#_ENREF_5)] |
| pZLL00 | Plasmid pZLB00 and pZP00 were incubated with integrase to generate a large plasmid which two *lacZα* reporter gene containing in frame inserted *attL* and *attR*, respectively. Then the plasmid was digested with BamHI and self-ligated. Then pZLL00 and pZLR00 were selected by NcoI digestion. | This work |
| pZLR00 | As described above. | This work |
| pZL5822 | *attL* and *attR* (φC31) in direct orientation after integration of pZL5805 [[4](#_ENREF_4)] and pSET152 [[3](#_ENREF_3)]. | [[5](#_ENREF_5)] |
| pTA0613 | PCR segment containing Apramycin resistant gene *aac(3)IV* flanked by *attB_6_*and *attP_13_*sites of φBT1was inserted into pMD19-T. | [[6](#_ENREF_6)] |
| pZLX01 | A 1191 bp overlapping PCR fragment (Primers X09/X10 and X11/X12) containing homologous arms of *gp3* (phage φC31) was cut with AseI/SphI, and pTA0613 was cut with NdeI/SphI, then the two fragments were ligated. | This work |
| pZLX02 | A 1045 bp PCR fragment (primers X13/X14) containing *Chl^R^* gene flanked by FRT sites, cut with NdeI/EcoRVand ligated into pZLX01 | This work |
| pZLX03 | The *gp3* gene (phage φBT1) from pET28-Bxis cut with NdeI/XhoI and ligated into pZLX02 | This work |
| pKD46 | Red recombination system, *Amp^R^*. | [[7](#_ENREF_7)] |
| pKD3 | Chloroamphenicol resistant gene flanked by FRT sites,*Amp^R^* and *Chl^R^*. | [[7](#_ENREF_7)] |

**Table S2.** Sequences of oligonucleotides.

| Names | Descriptions | Sequences |
| --- | --- | --- |
| X01 | *gp3*-φC31 | 5' - catatggcgaagcgttcgatc - 3' |
| X02 | *gp3*-φC31 | 5' - ctcgagactagtcggcaatcgcgtc - 3' |
| X09 | 5’-arm of *gp3*-φC31 | 5' - actcacattaatccttcacttgcgtcagac - 3' |
| X10 | 5’-arm of *gp3*-φC31 | 5' - gatatctttcgctagccatatgtgctcccaaagtgtcggg - 3' |
| X11 | 3’-arm of *gp3*-φC31 | 5' - tatggctagcgaaagatatcatcacggttctgaagtcg - 3' |
| X12 | 3’-arm of *gp3*-φC31 | 5' - tatggctagcgaaagatatcatcacggttctgaagtcg - 3' |
| X13 | *Chl^R^*gene from pKD3 | 5' - tatggctagcgaaagatatcatcacggttctgaagtcg - 3' |
| X14 | *Chl^R^*gene from pKD3 | 5' - tatggctagcgaaagatatcatcacggttctgaagtcg - 3' |
| ZL93 | 5-FAM labeled PCR primer | 5' - atgcagctggcacgacag - 3' (5-FAM labeled) |
| ZL95 | Overlapping, *attB_212,_ attL_306_* | 5' - atgcagctggcacgacagggaacaaaagctggag - 3' |
| ZL80 | *attB_212_*,*attR_153_* | 5' - gagcgcgcgtaatacgac - 3' |
| ZL94 | Overlapping, *attP_247,_ attR_153_* | 5' - atgcagctggcacgacagcagacgtttcgggtgctg - 3' |
| ZL82 | *attP_247_* | 5' - agcgcaacgcaattaatg - 3' |
| ZL88 | *attL_306_* | 5' - tcggtcgacacgtctgag - 3' |


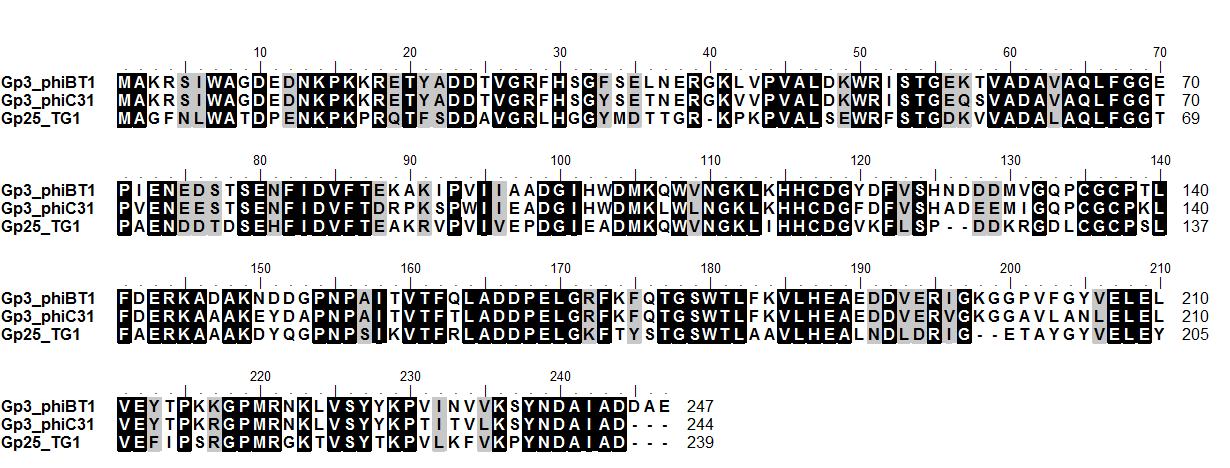


**Figure S1**. Amino acid sequence alignment of the putative Xis proteins from phages φBT1, φC31 and TG1. The alignment was performed using ClustalW. Gp3 sequences are of φBT1 (accession number AJ550940.2) and φC31 (accession number AJ006589.2) which showed 85% identity; Gp3 of φBT1 showed 62% identity with Gp25 of TG1 (accession number JX182372.1); and Gp3 of φC31 showed 60% identity with Gp25 of TG1 in BLASTP analyses.


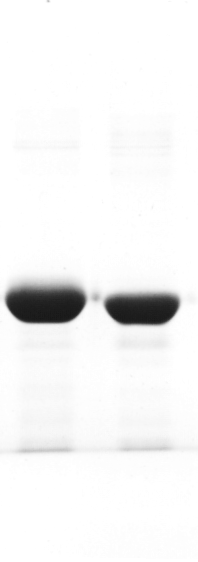

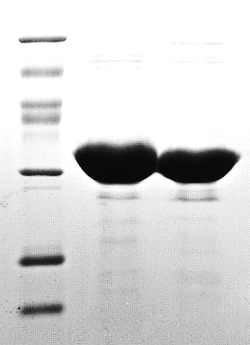


Gp3

φBT1 φC31

M

KDa

97

66

44

29

20

**Figure S2**. Purification of Gp3 of φBT1 and φC31 expressed in *E.coli*. Details of protein expression and purification were described in Materials and Methods, and the purified Gp3 was analyzed by 15% SDS-PAGE. The predicted sizes of 6×His-tagged proteins were 29.8 KDa (φBT1) and 29.6 KDa (φC31) respectively.

A B


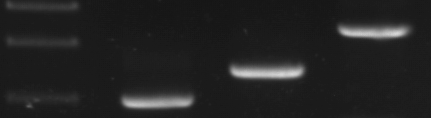


M123

3.3

2.7

1.8

Kbp


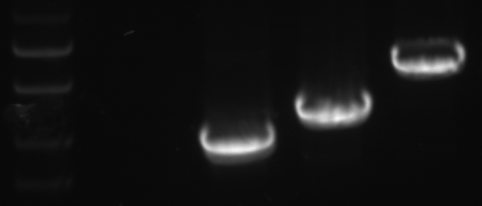


3.3

2.7

1.8

1.4

Kbp

MC123

**Figure S3**. PCR identification of the mutants.

**(A)** Identification of the replacement of *gp3*-φC31 gene. Plasmids φXD101, φXD101(X02) and φXD101(X03) were used as templates for PCR amplification with primers X09/X12; the product sizes were predicted as 1849 bp (φXD101, lane 1), 2219 bp (φXD101 (X02), lane 2) and 2924 bp (φXD101 (X03), lane 3).

**(B)** PCR identification of positive exconjugants of wild-type *S.co* J1929 and harbouring φXD101, φXD101(X02) or φXD101(X03). Primers used and the predicted product sizes were as described in (A).

**Supplementary References**

1. MacNeil DJ, Gewain KM, Ruby CL, Dezeny G, Gibbons PH, et al. (1992) Analysis of *Streptomyces avermitilis* genes required for avermectin biosynthesis utilizing a novel integration vector. Gene 111: 61-68.

2. Cowlishaw DA, Smith MC (2001) Glycosylation of a *Streptomyces coelicolor* A3(2) cell envelope protein is required for infection by bacteriophage φC31. Mol Microbiol 41: 601-610.

3. Kieser T, Bibb MJ, Buttner MJ, Chater KF, Hopwood DA (2000) Practical *Streptomyces* Genetics. Norwich, United Kingdom: The John Innes Foundation.

4. Zhang L, Ou XJ, Zhao GP, Ding XM (2008) Highly efficient *in vitro* site-specific recombination system based on *Streptomyces* phage φBT1 integrase. JBacteriol 190: 6392-6397.

5. Zhang L, Wang L, Wang J, Ou X, Zhao G, et al. (2010) DNA cleavage is independent of synapsis during *Streptomyces* phage φBT1 integrase-mediated site-specific recombination. J Mol Cell Biol 2: 264-275.

6. Zhang L, Zhao GP, Ding XM (2011) Tandem assembly of the epothilone biosynthetic gene cluster by *in vitro* site-specific recombination. Sci Rep 1:141.

7. Datsenko KA, Wanner BL (2000) One-step inactivation of chromosomal genes in *Escherichia coli* K-12 using PCR products. Proc Natl Acad Sci USA 97: 6640-6645.
